# Cross-Neutralization of Emerging SARS-CoV-2 Variants of Concern by Antibodies Targeting Distinct Epitopes on Spike

**DOI:** 10.1128/mBio.02975-21

**Published:** 2021-11-16

**Authors:** Siriruk Changrob, Yanbin Fu, Jenna J. Guthmiller, Peter J. Halfmann, Lei Li, Christopher T. Stamper, Haley L. Dugan, Molly Accola, William Rehrauer, Nai-Ying Zheng, Min Huang, Jiaolong Wang, Steven A. Erickson, Henry A. Utset, Hortencia M. Graves, Fatima Amanat, D. Noah Sather, Florian Krammer, Yoshihiro Kawaoka, Patrick C. Wilson

**Affiliations:** a University of Chicago Department of Medicine, Section of Rheumatology, Chicago, Illinois, USA; b Influenza Research Institute, Department of Pathobiological Sciences, School of Veterinary Medicine, University of Wisconsin—Madison, Madison, Wisconsin, USA; c Committee on Immunology, University of Chicago, Chicago, Illinois, USA; d UW Health Clinical Laboratories, University of Wisconsin Hospital and Clinics, Madison, Wisconsin, USA; e Department of Pathology and Laboratory Medicine, University of Wisconsin—Madison, Madison, Wisconsin, USA; f Department of Microbiology, Icahn School of Medicine at Mount Sinai, New York, New York, USA; g Center for Global Infectious Disease Research, Seattle Children’s Research Institute, Seattle, Washington, USA; h Department of Pediatrics, University of Washington, Seattle, Washington, USA; i Department of Global Health, University of Washington, Seattle, Washington, USA; j Division of Virology, Department of Microbiology and Immunology, Institute of Medical Science, University of Tokyo, Tokyo, Japan; Harvard School of Public Health

**Keywords:** SARS-CoV-2, humoral immunity, immune memory, infectious disease, monoclonal antibodies, single cell, variants of concern

## Abstract

Several severe acute respiratory syndrome coronavirus 2 (SARS-CoV-2) variants have arisen that exhibit increased viral transmissibility and partial evasion of immunity induced by natural infection and vaccination. To address the specific antibody targets that were affected by recent viral variants, we generated 43 monoclonal antibodies (mAbs) from 10 convalescent donors that bound three distinct domains of the SARS-CoV-2 spike. Viral variants harboring mutations at K417, E484, and N501 could escape most of the highly potent antibodies against the receptor binding domain (RBD). Despite this, we identified 12 neutralizing mAbs against three distinct regions of the spike protein that neutralize SARS-CoV-2 and variants of concern (VOCs), including B.1.1.7 (alpha), P.1 (gamma), and B.1.617.2 (delta). Notably, antibodies targeting distinct epitopes could neutralize discrete variants, suggesting that different variants may have evolved to disrupt the binding of particular neutralizing antibody classes. These results underscore that humans exposed to the first pandemic wave of prototype SARS-CoV-2 possess neutralizing antibodies against current variants and that it is critical to induce antibodies targeting multiple distinct epitopes of the spike that can neutralize emerging variants of concern.

## INTRODUCTION

The emergence of novel circulating severe acute respiratory syndrome coronavirus 2 (SARS-CoV-2) variants of concern (VOCs) has recently proven to undermine the protective effects of infection- and vaccination-induced humoral immunity (1–4). All approved vaccines against SARS-CoV-2 drive a neutralizing antibody response against the spike protein, the major target of neutralizing antibodies elicited by natural infection (3, 5). However, protective humoral immunity against the spike protein induced by vaccination or infection with the original wild-type (WT) virus may be attenuated due to the widespread circulation of variants (2). The first reported mutation of the SARS-CoV-2 spike protein, D614G, arose in the C-terminal domain (CTD) and evolved due to increased stability of the spike rather than a mutation to escape host immunity (6). More recently, mutations have arisen within the receptor binding domain (RBD), the N-terminal domain (NTD) of S1, and S2 that have resulted in the emergence of several circulating viral variants that are rapidly becoming the dominant strains around the globe (2). The B.1.1.7 lineage or alpha VOC, first found in the United Kingdom, has been reported to have >50% increased transmissibility among humans (7–10). Of the greatest concern is the substitution at position 484 in the RBD, which is exclusively shared by the VOCs, variants of interest (VOIs), and variants under monitoring (VUMs) originally identified in South Africa (B.1.351 [beta]), Brazil (P.1 [gamma]), Texas (R.1), Colombia (B.1.621 [mu]), New York (B.1.526 [iota]), and India (B.1.617.1 [kappa]) (2, 3, 11–15). VOCs possessing a mutation at E484, either E484K or E484Q, can partially evade neutralizing humoral immunity induced by either natural infection or vaccination and, in rare cases, can lead to reinfection or infection, respectively (11–13, 16–18). Other emerging variants have acquired a mutation of L452R within the RBD, which is found in B.1.1.298, a variant capable of interspecies transmission between humans and minks, and B.1.427/B.1.429 (epsilon) isolated in southern California (19). Moreover, B.1.617.1 (kappa) found in India possesses both L452R and E484Q mutations within the RBD (15, 20). The most recent VOC, B.1.617.2 (delta), is responsible for a surge in both cases and fatalities in several countries, especially where vaccination rates are low (4, 21–23). Intriguingly, the B.1.617 lineages contain P681R, a mutation that enhances and accelerates viral fusion (24) and is also present in the dominant variant in Uganda, A.23.1 (25). Thus, understanding the impact of these various mutations on the neutralization capacity of antibodies elicited by current vaccine formulations or natural exposure to WT SARS-CoV-2 is urgently needed to lay the foundation for next-generation vaccine strategies against SARS-CoV-2 variants.

Here, we report that natural WT SARS-CoV-2 infection induces memory B cells expressing potently neutralizing antibodies against VOCs. Moreover, natural WT infection largely induced antibodies against spike epitopes outside the RBD, most of which were non-neutralizing against the WT and VOCs. Additionally, RBD binding antibodies could be categorized into 3 distinct classes based on their binding profiles against RBD mutant constructs. We identified VOC-neutralizing antibodies against three distinct regions of the spike protein, including two epitopes on the RBD and one epitope in the NTD. Together, our study identifies that natural WT infection induces memory B cells that can produce neutralizing antibodies against recent SARS-CoV-2 VOCs and have the potential to be recalled by vaccination.

## RESULTS

### Convalescent-phase sera have reduced antibody titers but retain neutralization capabilities against circulating SARS-CoV-2 VOCs.

**FIG 1:**
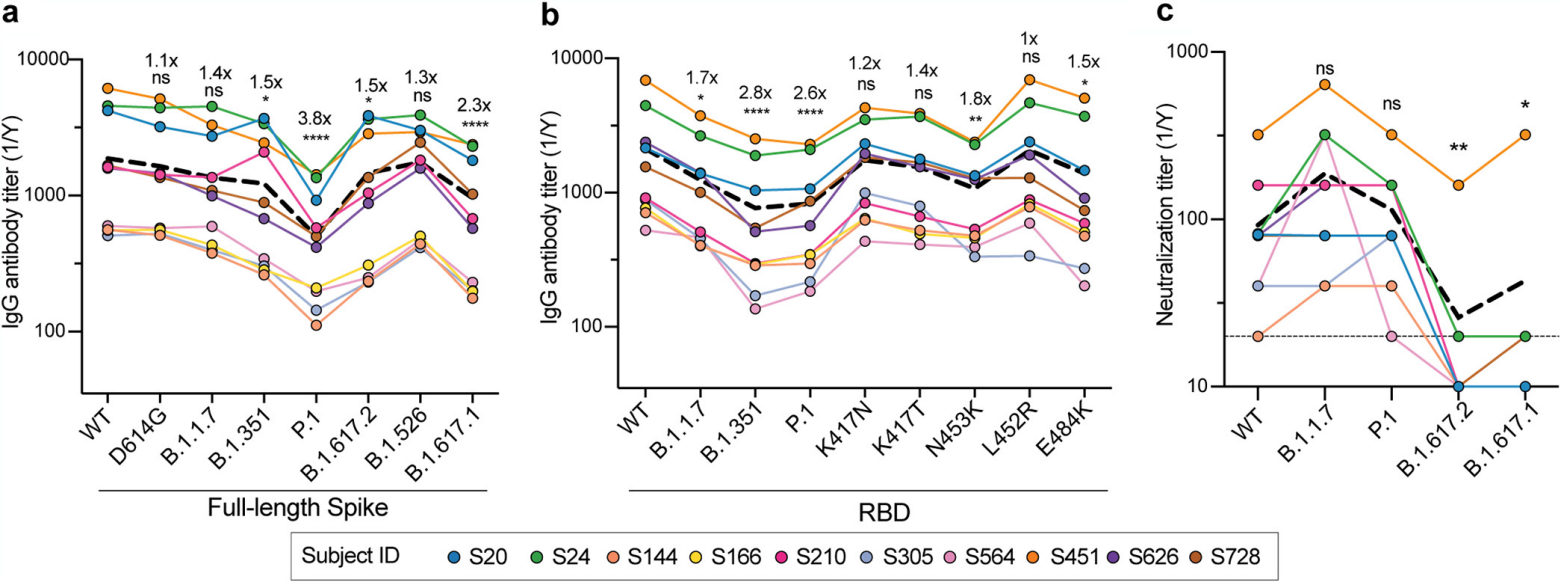
Analyses of serum antibody responses in COVID-19 convalescent individuals. (a and b) Total IgG endpoint antibody titers from 10 convalescent subjects against SARS-CoV-2 full-length spike variants (a) and RBD recombinant antigens (b). The dashed line is the mean IgG titer. (c) Neutralization titers from 10 convalescent donors against WT SARS-CoV-2, B.1.1.7, P.1, B.1.617.2, and B.1.617.1. The dashed line represents the mean neutralization titer. Data in panels a to c were analyzed using nonparametric Friedman’s test with Dunnett’s multiple-comparison test. Fold changes in relative mAb binding to variants or mutants compared to the WT in panels a and b are indicated above the statistical asterisks. ns, not significant.

To investigate whether antibodies from subjects naturally infected with WT SARS-CoV-2 lost binding or neutralization activity against VOCs, we collected blood samples from 10 convalescent donors at a median of 49 days after symptom onset (26, 27) (see Table S1 in the supplemental material) for an in-depth analysis of the specificity of individual memory B cells. As an initial estimate of antibody activity from these patients, serum antibody reactivity was measured by comparing reactivities to WT trimeric SARS-CoV-2 spike and spike proteins from the D614G, B.1.1.7, B.1.351, P.1, B.1.617.2, B.1.526, and B.1.617.1 variants. While serum antibody IgG titers from these 10 patients against WT and D614G spike antigens were similar, titers were significantly reduced against the spike proteins of B.1.1.7 (1.4-fold), B.1.351 (1.5-fold), P.1 (3.8-fold), B.1.617.2 (1.5-fold), B.1.526 (1.3-fold), and B.1.617.1 (2.3-fold) relative to the WT spike protein (Fig. 1a). Similarly, IgG titers against the RBDs of B.1.1.7 (1.7-fold), B.1.351 (2.8-fold), and P.1 (2.6-fold) were reduced compared to those against the WT RBD. However, we noted that there was less than a 2-fold decrease in antibody binding against single mutants of the RBD (Fig. 1b). Despite reductions in serum binding activity, the sera retained similar neutralizing titers against the WT and the B.1.1.7 and P.1 SARS-CoV-2 variants. However, we found a significant reduction in neutralization against B.1.617.1 and B.1.617.2 compared to the WT (Fig. 1c). Although antibody titers were lower against the VOCs and VUMs, these data indicate that serum antibodies elicited by natural WT infection were able to neutralize B.1.1.7, P.1, and WT viruses equally, while most donors lost neutralizing potential against B.1.617 lineage viruses.

10.1128/mBio.02975-21.4 TABLE S1 COVID-19 convalescent subjects. Responder group and severity were categorized in a previous study (26). Download Table S1, DOCX file, 0.03 MB. Copyright © 2021 Changrob et al. 2021 Changrob et al. https://creativecommons.org/licenses/by/4.0/ This content is distributed under the terms of the Creative Commons Attribution 4.0 International license.

### Generation of mAbs against distinct domains of the SARS-CoV-2 spike.

**FIG 2:**
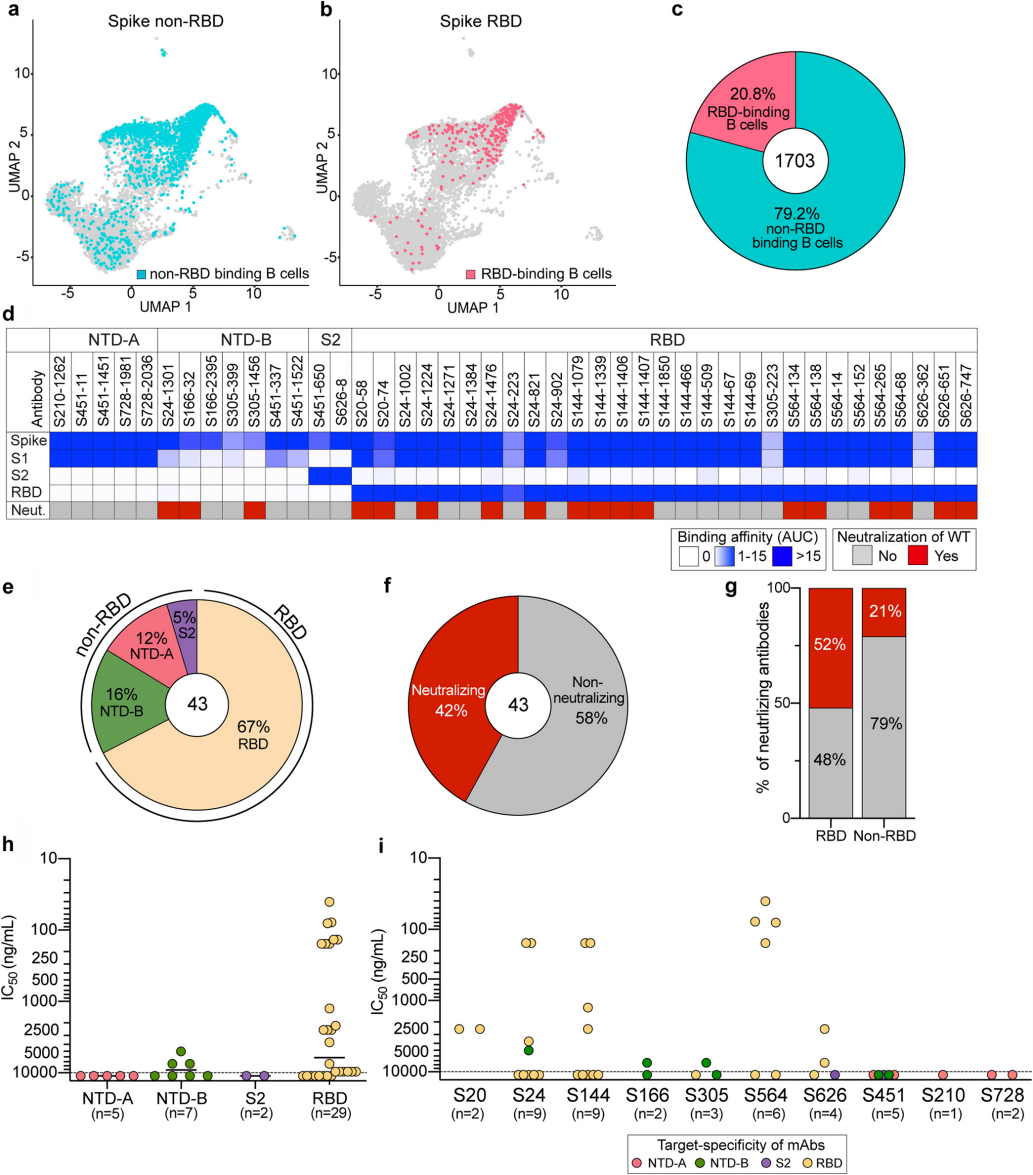
Characterization of spike-reactive mAbs. (a and b) Uniform manifold approximation and projection (UMAP) of SARS-CoV-2 spike non-RBD binding (a) and spike RBD binding (b) B cells isolated from PBMCs of 10 convalescent subjects. (c) Proportion of spike non-RBD- and spike RBD-specific binding B cells. The number in the center of the pie chart indicates the number of antigen-specific binding B cells. (d) mAbs generated from selected B cells (*n* = 43) were tested for binding to full-length spike, S1, S2, and the RBD and neutralization potential against WT SARS-CoV-2. Binding data are represented as areas under the curve (AUC). Neutralizing activities of <10,000 ng/ml are considered neutralizing. (e and f) Pie charts of mAb domain specificity (e) and neutralizing capability (f). The numbers in the center of the pie graphs indicate the number of antibodies tested. (g) Comparison of neutralizing capabilities of mAbs targeting the spike RBD and spike non-RBD. (h and i) IC_50_s of the neutralization potencies of spike-reactive antibodies against WT virus based on domain specificity (h) and by subject (i). The mean in panel h is indicated as a solid line. Data in panels h and i are colored based on domain specificity, and the dashed lines shown in panels h and i indicate the limit of detection (10,000 ng/ml). Data in panels d to i are representative of results from two independent experiments performed in duplicate. Genetic characterization of each mAb is further detailed in Table S2 in the supplemental material.

We next sought to determine the specificities of antibodies that could cross-neutralize these viral variants by generating monoclonal antibodies (mAbs) from spike binding B cells isolated from 10 convalescent subjects, collected between April and July 2020 (26, 27). We sorted B cells binding to spike and/or RBD fluorophore- and oligonucleotide-conjugated probes and performed single-cell RNA sequencing (RNA-seq) and B cell receptor sequencing. As the antigen probes included a DNA oligonucleotide sequence, we were able to track the antigen specificity of isolated B cells. In total, we obtained 1,703 paired immunoglobulin heavy and light chains from non-RBD and RBD binding B cells specific for the spike. Overall, the percentage of spike non-RBD binding B cells was 4-fold higher than that of RBD binding B cells (Fig. 2a to c), indicating that natural WT infection preferentially induced B cell responses to epitopes on the spike outside the RBD (28, 29). Overall, B cells targeting the RBD or epitopes outside the RBD utilized similar V(D)J genes, had overlapping heavy and light chain pairings, and possessed similar numbers of mutations and complementarity-determining region 3 (CDR3) lengths (Fig. S1a to i).

10.1128/mBio.02975-21.1 FIG S1 mAb genetic, somatic hypermutation, and CDR3 length features. (a to d) Distribution of the V gene usage of spike non-RBD and spike RBD antibodies for all paired heavy (a and c) and light (b and d) chains. The percentage shown indicates the proportion of the top 3 utilized genes. (e) Clonal relationships between heavy and light chain variable gene loci of spike non-RBD- and spike RBD-specific antibodies. Connecting lines represent the pairing of heavy and light chains of antibody clones specific to the spike non-RBD (blue) or RBD (red) and antibody clones shared by both groups (purple). (f and g) Comparison of the numbers of somatic hypermutations of heavy (f) and light (g) chains of spike non-RBD and spike RBD binding B cells. (h and i) Complementarity-determining region 3 (CDR3) amino acid lengths for heavy (h) and light (i) chains of spike non-RBD and spike RBD binding B cells. The median is indicated as a line in the box-and-whisker graph. Each dot represents an individual antibody with a range from the minimum to maximum values. Data in panels f to i were analyzed using a Mann-Whitney nonparametric test. Download FIG S1, DOCX file, 1.3 MB. Copyright © 2021 Changrob et al. 2021 Changrob et al. https://creativecommons.org/licenses/by/4.0/ This content is distributed under the terms of the Creative Commons Attribution 4.0 International license.

10.1128/mBio.02975-21.5 TABLE S2 Characteristics of SARS-CoV-2 spike binding mAbs. Cross-neutralizing mAbs against the WT, B.1.1.7, and P.1 or B.1.617.2 are in boldface type. Download Table S2, DOCX file, 0.03 MB. Copyright © 2021 Changrob et al. 2021 Changrob et al. https://creativecommons.org/licenses/by/4.0/ This content is distributed under the terms of the Creative Commons Attribution 4.0 International license.

Based on the acquired antibody sequences and probe binding intensities, we generated 43 mAbs from all 10 donors specific for the WT spike protein (Table S2). To investigate specific domain targeting, mAbs were tested for binding to the RBD and monomeric S1 and S2 recombinant spike antigens. Based on binding to these discrete antigens, spike-reactive mAbs were categorized into 4 groups: NTD-A-reactive mAbs (*n* = 5) that bound strongly to S1 but not the RBD, NTD-B-reactive mAbs (*n* = 7) that weakly bind S1 but not the RBD, S2-reactive mAbs (*n* = 2), and RBD-reactive mAbs (*n* = 29) (Fig. 2d and e). Additionally, NTD-A- and NTD-B-classified antibodies targeted distinct epitopes as shown by a competition enzyme-linked immunosorbent assay (ELISA) (Fig. S2a). We further determined whether antibodies with different binding specificities differ in their neutralization capacities against WT SARS-CoV-2. Of the 43 mAbs, 18 (42%) were neutralizing. Notably, only mAbs binding the RBD and NTD-B were neutralizing, whereas all mAbs binding NTD-A and S2 were non-neutralizing (Fig. 2d to f). Moreover, 52% of RBD-targeting mAbs were neutralizing, with eight mAbs being potently neutralizing antibodies (50% inhibitory concentration [IC_50_] of <500 ng/ml) and three out of seven NTD-B mAbs having moderate neutralization potency (5,000 to 7,500 ng/ml) (Fig. 2g and h). Of the 10 convalescent donors, 7 had at least one neutralizing mAb among the antibodies cloned for this study, although the potencies of the mAbs varied by donor (Fig. 2i). Together, these data reveal that mAbs against the RBD are the predominant source of neutralizing antibodies induced by WT SARS-CoV-2 infection.

10.1128/mBio.02975-21.2 FIG S2 mAb binding competition by an ELISA and BLI and serum competition by an ELISA. (a) Competition ELISA of RBD mAbs or spike non-RBD mAbs with NTD-A (S451-11) and NTD-B (S305-1456) mAbs. (b) Competition ELISA of RBD mAbs of an undetermined class with class 2 mAbs (S144-1079 and S564-138) and a class 3 mAb (S24-821). (c) mAb binding competition by BLI of a class 2 mAb, S144-1406, with the other class 2 mAbs (*n* = 4) that did not neutralize P.1. (d) mAb binding competition by BLI between class 4 mAbs that utilized VH5-51 (S144-466, S144-509, S144, and S144-69) and CR3022. (e) EC_50_s of serum antibodies of 10 convalescent subjects competing with RBD-reactive mAbs for binding to RBD class 2, class 3, and class 3-like epitopes and NTD-reactive mAbs for binding to NTD-B epitopes. The dashed line represents the limit of detection. Data in panels a, b, and e are representative of results from two independent experiments performed in duplicate. Data in panel e were analyzed using nonparametric Friedman’s test with Dunn’s multiple-comparison test. Download FIG S2, DOCX file, 0.5 MB. Copyright © 2021 Changrob et al. 2021 Changrob et al. https://creativecommons.org/licenses/by/4.0/ This content is distributed under the terms of the Creative Commons Attribution 4.0 International license.

### Binding and neutralizing breadth of non-RBD spike antibodies.

**FIG 3:**
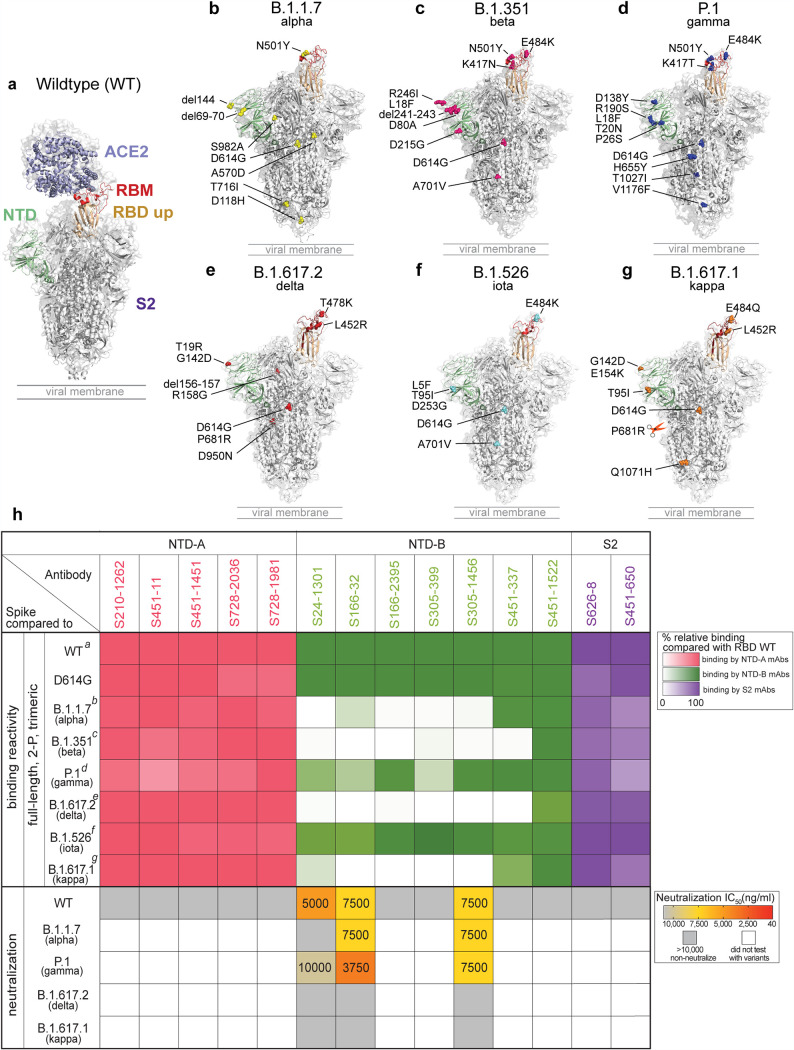
Binding breadth and neutralization of spike non-RBD mAbs. (a) Full-length spike protein binding to ACE2 (PDB accession number 7KJ2). (b to g) Locations of mutations found on B.1.1.7 (b), B.1.351 (c), P.1 (d), B.1.617.2 (e), B.1.526 (f), and B.1.617.1 (g) (modified from the structure under PDB accession number 6XM4). (h) Binding reactivity and neutralization capabilities 5 of NTA-A (pink)-, NTD-B (green)-, and S2 (purple)-reactive mAbs. The color gradients indicate the percentages of relative binding compared to WT spike. The neutralization potencies (IC_50_) of spike non-RBD mAbs against the WT and the B.1.1.7, P.1, B.1.617.2, and B.1.617.1 variants are indicated in nanograms per milliliter. The panel of SARS-CoV-2s is detailed in Table S4 in the supplemental material. Data in panel h are representative of results from two independent experiments performed in duplicate. Genetic information for each mAb can be found in Table S2.

To understand the effects of viral variants on mAb binding to epitopes on the spike outside the RBD, we tested non-RBD-targeting mAbs for binding to a panel of SARS-CoV-2 variants, including D614G and the emerging variants B.1.1.7, B.1.351, P.1, B.1.617.2, B.1.526, and B.1.617.1 (Fig. 3a to g). All non-RBD spike-reactive antibodies showed similar binding to the D614G spike. Furthermore, all mAbs targeting NTD-A and S2 maintained similar binding to the spike of the B.1.1.7, B.1.351, P.1, B.1.617.2, B.1.526, and B.1.617.1 variants (Fig. 3h). Although mAbs against NTD-A and S2 retain binding to VOCs, they are nonneutralizing, implying that NTD-A- and S2-reactive antibodies may provide limited immune pressure to mutate these epitopes. Of interest, NTD-B mAbs showed significantly reduced binding to the spike of B.1.1.7, B.1.351, B.1.617.2, and B.1.617.1 while showing similar binding to B.1.526 and a minor reduction in binding to the spike of P.1 (Fig. 3h). Two of the three neutralizing NTD-B binding mAbs (S166-32 and S305-1456), which were isolated from two different subjects, retained neutralization potential against B.1.1.7 and P.1 at moderate neutralizing potency (Fig. 3h). The third neutralizing NTD-B binding mAb (S24-1301) also had moderate neutralizing potency against the WT strain, with weak cross-neutralization activity against the P.1 variant and no neutralization activity against B.1.1.7, consistent with its binding profile (Fig. 3h). However, all three neutralizing NTD-B mAbs failed to neutralize B.1.617.1 and B.1.617.2. Together, our data indicate that antibodies against NTD-B show cross-neutralization capacity and thus may provide protection against some emerging VOCs such as B.1.1.7 and P.1. However, antibodies targeting the NTD-B epitope may be driving spike evolution, particularly the mutations and deletions found within B.1.1.7, B.1.351, B.1.617.1, and B.1.617.2, leaving the future of this epitope as a reliable target for cross-reactive antibodies uncertain.

10.1128/mBio.02975-21.7 TABLE S4 SARS-CoV-2 virus information and resource. Download Table S4, DOCX file, 0.03 MB. Copyright © 2021 Changrob et al. 2021 Changrob et al. https://creativecommons.org/licenses/by/4.0/ This content is distributed under the terms of the Creative Commons Attribution 4.0 International license.

### A subset of RBD-binding mAbs retains neutralization activity against VOCs.

**FIG 4:**
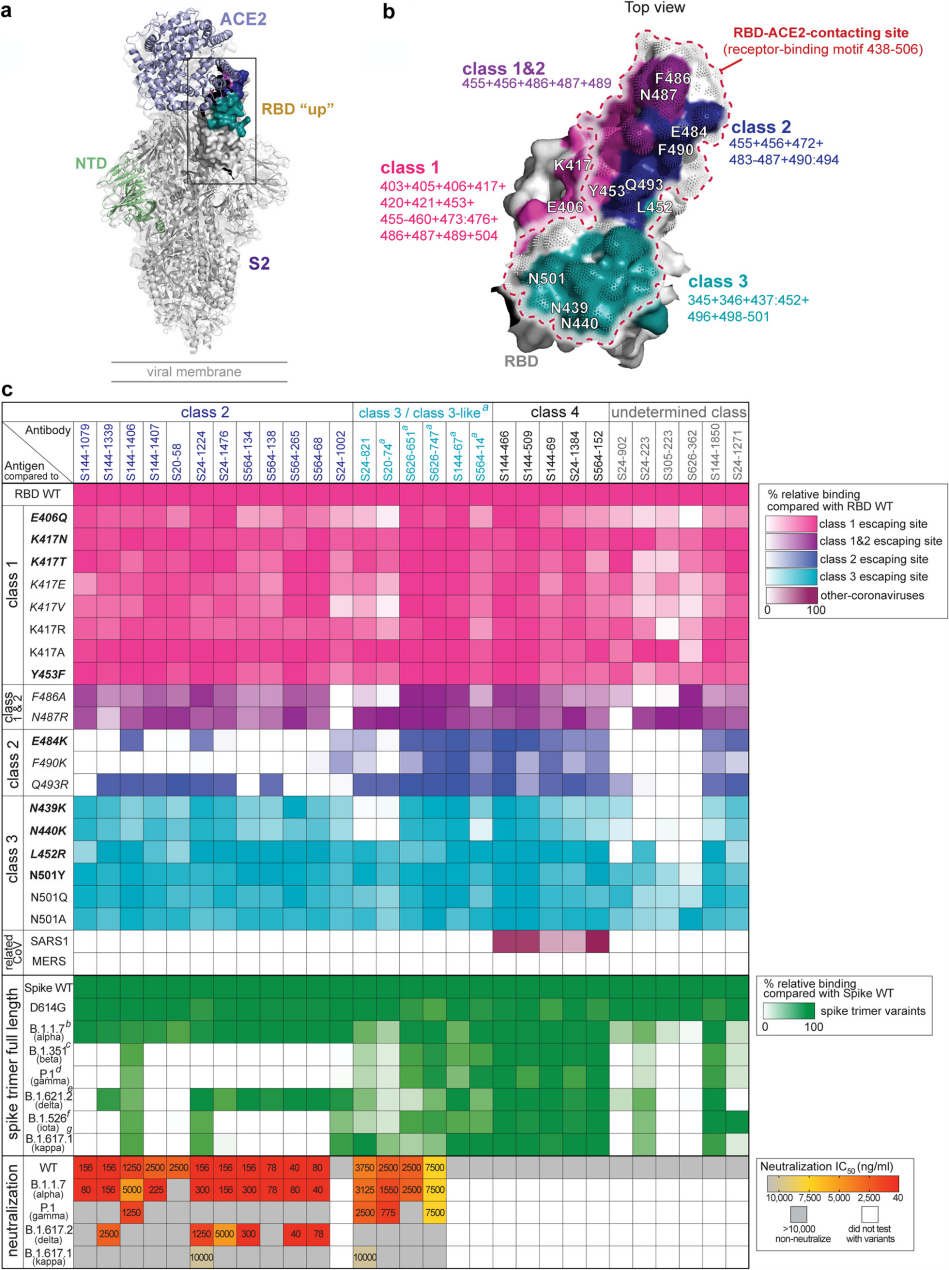
Binding and neutralization profiles of RBD binding mAbs against a panel of RBD escape mutants and variants. (a) Structural model of RBD “up” binding with ACE2 (PDB accession number 7KJ2) and RBD antibody classes and associated escape mutants. (b) RBD colored by antibody classes and associated mutations. Pink, class 1; purple, overlap of classes 1 and 2; blue, class 2; teal, class 3. (c) Heat map detailing the binding reactivity of RBD mAbs (*n* = 29) against single key escape sites for class 1, class 2, and class 3 antibodies; combinations of RBD mutants; and the RBDs from SARS-CoV-1 and MERS-CoV. *a* refers to class 3-like antibodies, which are defined by mAbs that compete with a class 3 mAb (see Fig. S2c in the supplemental material). *b* to *f* refer to mutations in the RBD of each full-length spike variant, B.1.1.7 with N501Y (*b*), B.1.351 with K417N:E484K:N501Y (*c*), P.1 with K417T:E484K:N501Y (*d*), B.1.617.2 with T478K:L452R (*e*), B.1.526 with E484K (*f*), and B.1.617.1 with L452R:E484Q (*g*). The panel of recombinant antigens in panel c is detailed in Table S3, including mutations found in circulating SARS-CoV-2 variants (boldface type), mutations that escape/reduce binding by polyclonal serum/potent neutralizing mAbs (italic type), mutations found in both circulating SARS-CoV-2 variants and the *in vitro* escape map (boldface and italic type), and artificial mutants at key contact residues of the RBD-ACE2 interaction (normal typeface). The neutralization potencies (IC_50_) of spike RBD mAbs against the WT and the B.1.1.7, P.1, B.1.617.2, and B.1.617.1 variants are indicated in nanograms per milliliter. The panel of SARS-CoV-2s is detailed in Table S4. Data in panel c are representative of results from two independent experiments performed in duplicate. Genetic information for each antibody can be found in Table S2.

Viral escape mutations occurring within the RBD may result in a reduction of the neutralization capacity of RBD-targeting antibodies (30–32). To understand the impacts of RBD mutations on mAb binding, we tested RBD-targeting mAbs for binding to RBD mutants that possessed a single mutation found in circulating SARS-CoV-2 VOCs, VOIs, VUMs and artificial mutants at key contact residues of the RBD-ACE2 interaction (30–35) as well as full-length (FL) spike constructs containing multiple mutations in the RBD (Table S3). In addition, we tested mAb binding to the RBDs of SARS-CoV-1 and Middle East respiratory syndrome coronavirus (MERS-CoV) to investigate cross-reactivity to other coronaviruses. Notably, RBD binding mAbs have been classified into four classes, classes 1 to 4 or receptor binding sites (RBSs) A to D, based on structural analysis and antibody binding features (36, 37). More recently, the classification of four key antigenic regions of the RBD can also be defined by determining the loss of binding to RBD mutants (class 1 to 3 epitopes) or binding to cryptic epitopes on the RBD that are conserved across SARS-CoV-1 and MERS-CoV RBDs (class 4 epitope) (Fig. 4a and b) (17, 30). Based on the binding profiles of class 1 to 4 binding mAbs, we were able to segregate 23 out of 29 mAbs into one of the four classes (Fig. 4c and Fig. S2b). Notably, no class 1 mAbs were found and six mAbs could not be classified as they either lost binding to multiple mutant classes or bound equally to all RBD mutants but did not bind to SARS-CoV-1 or MERS-CoV.

10.1128/mBio.02975-21.6 TABLE S3 Recombinant antigen information and resource. VOC, variant of concern; VUM, variant under monitoring. Download Table S3, DOCX file, 0.03 MB. Copyright © 2021 Changrob et al. 2021 Changrob et al. https://creativecommons.org/licenses/by/4.0/ This content is distributed under the terms of the Creative Commons Attribution 4.0 International license.

Class 2 RBD binding mAbs showed reduced binding to at least one of the RBD class 2 single escape mutants, notably E484K and F490K, and the majority of these mAbs lost binding to the RBD mutants found in B.1.351, P.1, B.1.526, and B.1.617.1 (Fig. 4c). Of the 12 class 2 mAbs, 11 were potently neutralizing against WT SARS-CoV-2. Of the neutralizing class 2 mAbs, all but one neutralized B.1.1.7 at concentrations comparable to those for neutralization of the WT strain. In contrast, six mAbs neutralized B.1.617.2 at a lower potency than for the WT and B.1.1.7. Seven of the class 2 mAbs retained their neutralization activity against at least two VOCs (Fig. 4c). Of note, 10 out of 11 neutralizing class 2 mAbs were unable to neutralize the variants that harbored a mutation at E484, P.1 and B.1.617.1. This is in line with previous studies that showed that the E484K and E484Q mutations are the key escaping residues responsible for neutralization resistance by P.1, P.2, B.1.351, and B.1.617.1 VOCs (2, 4, 38). Of the greatest interest, S144-1406, which retained binding to E484K and all spike variants, neutralized B.1.1.7 and P.1 variants with high neutralization potency. Similar to another E484K binder, S24-1224 neutralized three out of four VOCs tested, including B.1.617.1 (Fig. 4c). These data indicate that some class 2 antibodies can cross-neutralize VOCs. Additionally, the epitope targeted by S144-1406 partially overlapped the ones targeted by S24-1224 and other class 2 mAbs that failed to neutralize P.1 and B.1.617.1 (Fig. S2c), suggesting that class 2 mAbs target similar but slightly different RBD epitopes.

Only one mAb (S24-821) specifically lost binding to the class 3 mutants, particularly to N439K and N440K, which are associated with circulating SARS-CoV-2 variants (35, 39) and have been reported as *in vitro* escape sites for class 3 epitope binding mAbs (30, 31, 35) (Fig. 4b and Table S3). Moreover, we classified five more mAbs as class 3-like as they strongly competed for RBD binding with S24-821 but did not compete with class 2 mAbs (Fig. S2b). Importantly, all class 3 and class 3-like mAbs maintained binding to L452R, another mutation associated with class 3 antibodies that is present in B.1.427/B.1.429 (19, 40) and B.1.617 (20) variants (Fig. 4c). However, there was a 2- to 3-fold reduction of class 3 and class 3-like mAbs in binding against B.1.617.2 that carries T487K and L452R substitutions in the RBD region. Of the four neutralizing class 3 and class 3-like mAbs, all four retained neutralization activity against B.1.1.7, and three were neutralizing against P.1 (Fig. 4b). In contrast to class 2 mAbs, B.1.617.2 was resistant to all class 3-neutralizing mAbs. Only one mAb (S24-821) retained modest neutralization potency against B.1.617.1, indicating that antibodies binding class 3 epitopes could neutralize some VOCs even though they bound the L452R single mutation and all spike variants.

All of the mAbs that were categorized into class 4 (*n* = 5) maintained binding to all RBD mutants and spike variants and displayed cross-reactivity to the SARS-CoV-1 RBD. However, all class 4 mAbs were non-neutralizing against WT virus, suggesting that antibodies against this epitope are likely not strong drivers of antigenic drift. Notably, three antibodies in the class 4 group utilized the same heavy chain gene, VH5-51, as CR3022 and competed with CR3022 for binding to the RBD, indicating that the class 4 antibodies in our study likely target the same or a similar epitope as CR3022 (Table S2 and Fig. S2d). This is consistent with a previous study showing that CR3022 cross-reacts with SARS-CoV-1, suggesting that class 4 antibodies are common across subjects and studies (17, 30).

With the classification of mAbs against distinct epitopes, we next tested the relative abundances of serum antibodies against these distinct epitopes of the RBD and NTD by performing competition assays. Notably, donors had significantly higher titers of serum antibodies targeting class 3 (S24-821) and class 3-like (S20-74) epitopes, whereas subjects largely had undetectable titers against class 2 and NTD-B epitopes, suggesting that WT SARS-CoV-2 infection predominantly induces polyclonal antibodies targeting RBD class 3 epitopes that can neutralize the emerging VOCs B.1.1.7 and P.1 (Fig. S2e). These data are consistent with the observed anti-B.1.1.7 and anti-P.1 serum neutralizing titers shown in Fig. 1c, suggesting that the retention of serum neutralization activity could be due to abundant class 3 antibody responses. The loss of neutralization capabilities against B.1.617 lineage viruses may be due to insufficient levels of class 2 serum antibodies. A comparison of the neutralization capabilities of mAbs targeting different epitopes revealed that class 2 RBD-reactive mAbs were the most potently neutralizing, followed by mAbs targeting class 3 RBD epitopes and NTD-B (Fig. S3a). It is important to note that none of neutralizing mAbs induced by natural WT infection were able to neutralize all emerging SARS-CoV-2 variants. Nonetheless, we identified at least one mAb that could neutralize each VOC, suggesting that the convalescent donors generated a diverse cross-neutralizing antibody response (Fig. S3b). Therefore, antibodies targeting multiple epitopes on the spike are a valuable source of neutralizing antibodies against emerging VOCs. Additionally, we found that the majority of antibodies isolated from donors who had high antibody titers exhibited lower neutralizing potency than antibodies derived from donors who had lower serological titers and less severity (Fig. S3b and Table S1). However, there was no difference between high and low responders in generating cross-neutralizing antibodies against VOCs and VUMs. Moreover, the cross-neutralizing RBD-targeting mAbs used V(D)J gene features similar to those for other previously reported RBD binding mAbs (Table S3) (41–43). However, the mAbs in our studies utilized distinct heavy and light chain pairings, indicating that these clones are not public with other known neutralizing SARS-CoV-2 antibodies. Despite this, our data indicate that cross-neutralizing antibodies use a diverse antibody repertoire against multiple distinct epitopes. Therefore, driving a polyclonal antibody response against these three epitopes may provide cross-neutralizing protection against existing and future variants.

10.1128/mBio.02975-21.3 FIG S3 Comparison of neutralization potencies of SARS-CoV-2-neutralizing mAbs. (a) Neutralization potencies (IC_50_) of RBD binding mAbs, class 2 and 3 mAbs, and NTD-B binding mAbs against WT SARS-CoV-2. (b) Neutralization potency of each mAb from each subject against WT SARS-CoV-2 (red), B.1.1.7 (blue), P.1 (yellow), B.1.617.2 (green), and B.1.617.1 (plum). Each dot indicates one mAb. mAbs that neutralize VOCs are in boldface type. Data in panels a to c are representative of results from two independent experiments performed in duplicate. Data in panel a were analyzed using a Mann-Whitney nonparametric test. Download FIG S3, DOCX file, 0.2 MB. Copyright © 2021 Changrob et al. 2021 Changrob et al. https://creativecommons.org/licenses/by/4.0/ This content is distributed under the terms of the Creative Commons Attribution 4.0 International license.

## DISCUSSION

Our study shows that WT SARS-CoV-2 convalescent individuals possess antibodies that can effectively cross-neutralize emerging VOCs, with cross-neutralizing antibodies targeting multiple epitopes of the spike protein. In total, we identified 12 mAbs that potently neutralize currently circulating VOCs, including B.1.1.7, the alpha variant, which has been reported to be more infectious (8, 19); P.1, the gamma variant, which partially escapes both natural and vaccine-induced humoral immunity (2, 12, 44); and B.1.617.2, the delta variant, which is more transmissible than the alpha variant, has led to a surge of more hospitalizations in India, and can evade partial immunity induced by one vaccine dose (4, 15, 23). Convalescent subjects in our cohort had sufficient serum titers to neutralize both B.1.1.7 and P.1 but not B.1.617, suggesting that the cross-neutralizing mAbs identified in this study may play an important role in polyclonal neutralization for some of the VOCs.

Using high-throughput antigen probing at the single-B-cell level, we found that B cells isolated from convalescent subjects largely targeted non-RBD epitopes rather than potently neutralizing epitopes on the RBD. Similarly, mRNA vaccines also largely induce antibodies against non-neutralizing epitopes, suggesting that epitopes outside the RBD are immunodominant (3, 45). Despite this, vaccination has been shown to induce cross-neutralizing antibodies (1), suggesting that both natural WT infection and currently approved vaccines can elicit protective humoral immunity against emerging variants. As we identified 12 antibodies cross-neutralizing to VOCs derived from seven different convalescent coronavirus disease 2019 (COVID-19) donors, our study suggests that most people generate a cross-neutralizing antibody response. Notably, these antibodies largely target three distinct epitopes, including two sites on the RBD and one on the NTD. Several recent studies have demonstrated that antibodies against the NTD and S2 are neutralizing (46–48). Although the anti-S2 mAbs identified in our study were non-neutralizing, S2 binding antibodies exhibit broad reactivity with spike proteins from SARS-CoV-2 variants, related betacoronaviruses such as SARS-CoV-1 and MERS-CoV, and distantly related endemic coronaviruses. Moreover, anti-spike serum antibodies can mediate protection via Fc-mediated functions, suggesting that a combination of neutralizing antibodies and polyfunctional antibodies will provide optimal protection against infection with variants of SARS-CoV-2 (49).

Our study also showed that anti-RBD mAbs are primarily class 2 mAbs, consistent with other reports (30, 37, 42, 50). The majority of the class 2 mAbs retained their neutralization activity against B.1.1.7 and B.1.617.2 but were largely non-neutralizing against P.1, suggesting that class 2 mAbs may have driven the evolution of P.1 mutants. In contrast, neutralizing class 3 mAbs retained their neutralization activity against both B.1.1.7 and P.1 but did not neutralize the B.1.617 variants. Notably, none of the neutralizing mAbs could cross-neutralize B.1.1.7, P.1, and B.1.617.2, the most prevalent VOCs as of writing. Therefore, vaccination approaches to increase the affinity and frequencies of antibodies to the S1 domain may enhance the breadth of protection against emerging SARS-CoV-2 VOCs, including epitopes on the RBD and NTD. It is likely that targeting multiple epitopes will provide optimal protection to avoid the generation of escape mutants that can evade antibodies against any one epitope. Moreover, vaccinating previously infected subjects has been shown to substantially improve neutralization titers (3) and may allow refinement of memory B cells against neutralizing epitopes.

In conclusion, our study shows that SARS-CoV-2 infection induces cross-neutralizing immunity against circulating VOCs, which is likely attributed to polyclonal antibodies targeting multiple epitopes of the spike protein. This work emphasizes the need for the induction of cross-neutralizing antibodies that bind distinct sites on the spike with various mechanisms that can synergize to provide protection against SARS-CoV-2 variants as well as limit the virus from escaping any single antibody target.

## MATERIALS AND METHODS

### Study cohort and spike-specific B cell sorting.

All studies were performed with the approval of the University of Chicago institutional review board (IRB20-0523), the University of Chicago, and the University of Wisconsin—Madison. Informed consent was obtained after the research applications and possible consequences of the studies were disclosed to study subjects. This clinical trial was registered at ClinicalTrials.gov under identifier NCT04340050, and clinical information for patients included in the study is detailed in Table S1 in the supplemental material. The details of peripheral blood mononuclear cell (PBMC) collection from leukoreduction filters were described previously (27). For spike-specific B cell sorting, PBMCs were thawed in a 37°C water bath, and B cells were enriched using a human pan-B cell EasySep enrichment kit (Stemcell). B cells were stained with anti-CD19-phycoerythrin (PE)-Cy7 (BioLegend), anti-CD3-BV510 (BD Biosciences), and antigen probes (PE) for 30 min on ice in 1× phosphate-buffered saline (PBS) supplemented with 0.2% bovine serum albumin (BSA) and 2 mM Pierce biotin. Probe generation was performed as previously described (27). Cells were subsequently washed with 1× PBS with 0.2% BSA and stained with Live/Dead BV510 (Thermo Fisher) in 1× PBS for 15 min. Cells were washed again and resuspended at a maximum of 4 million cells/ml in 1× PBS supplemented with 0.2% BSA and 2 mM Pierce biotin for downstream cell sorting using the MACSQuantTyto cartridge sorting platform (Miltenyi). Viable/CD19^+^/antigen-PE-positive cells were sorted as probe positive. Cells were then collected from the cartridge sorting chamber and used for downstream processing with a chromium controller (10X Genomics).

### Single-cell RNA-seq and B cell receptor sequencing.

The human B cell V(D)J, 5′ gene expression, feature barcode libraries were prepared according to the manufacturer’s instructions. Libraries were pooled and sequenced using an Illumina NextSeq550 or an Illumina NextSeq 500 platform at the University of Chicago. Cell Ranger (version 3.0.2) was used to perform raw sequence processing, sample demultiplexing, barcode processing, single-cell 5′ transcript counting, and B cell receptor repertoire sequence assembly. The reference genome assembly for the transcriptome is GRCh38-1.2.0, and the reference genome assembly for V(D)J is cellranger-vdj-GRCh38-alts-ensembl-2.0.0. The data obtained from Cell Ranger were subsequently used for downstream analysis using the Seurat toolkit (version 3.2.0) (an R package for transcriptome, cell surface protein, and antigen probe analyses) (51) and IgBlast (version 1.15) for immunoglobulin gene analysis (52). Cell quality control (QC), normalization, data scaling, linear dimensional reduction, clustering, differential expression analysis, batch effect correction, and data visualization were performed using Seurat (version 3.2.0). QCs of cells were performed further to exclude cells with <200 and >2,500 detected genes and cells expressing a high percentage of mitochondrial genes. Transcriptome RNA data were analyzed using conventional log normalization. We performed a principal-component analysis (PCA) and used the top 15 principal components (PCs) for linear dimensional reduction and clustering. Only filtered, high-quality cells were clustered in this analysis using the Louvain algorithm implemented in Seurat under a resolution of 0.6 for clustering. Batch effects across different data sets were normalized using an Anchor method implemented in Seurat.

### Monoclonal antibody production.

B cells were selected for mAb generation based on antigen probe intensity visualized by JMP Pro 15, as previously described (27). Antibody heavy and light chain genes obtained by 10X Genomics V(D)J sequencing analysis were synthesized by Integrated DNA Technologies. The synthesized fragments for heavy and light chains with 5′ and 3′ Gibson overhangs were then cloned into human IgG1 and human kappa or lambda light chain expression vectors by Gibson assembly as previously described (53). The heavy and light chains of the corresponding mAb were cotransfected into HEK293T cells. After 4 days, mAbs secreted into the medium supernatant were harvested and purified using protein A-agarose beads (Thermo Fisher).

### Recombinant proteins.

The recombinant WT SARS-CoV-2 full-length (FL) spike, D614G FL spike, WT RBD, K417T/R/A RBD, N501Q/A RBD, and SARS-CoV-1 RBD and MERS-CoV were generated in-house either by using a gBlock fragment synthesized by Integrated DNA Technologies or by performing single-site mutagenesis and expressed by Expi293F cells (Thermo Fisher). The recombinant FL spikes derived from variants B.1.1.7, B.1.351, P.1, B.1.617.2, B.1.526, and B.1.617.1 were kindly provided by the Noah Sather laboratory at Seattle Children’s Research Institute. The recombinant RBDs found in VOCs, B.1.351 or P.1 variants, and RBDs with single or multiple mutations (N439:Y453F, E406Q, K417E, K417V, Y453F, F486A, N487R, F490K, Q493R, N439K, N440K, and N501Y) were generously provided by the Krammer laboratory at the Icahn School of Medicine at Mount Sinai. The recombinant S1 and S2 subunits and RBDs with single mutations of K417N, E484K, and L452R were obtained from Sino Biological. The protein sequences and resources for each antigen are listed in Table S3.

### Virus neutralization assay.

Virus neutralization assays were performed with different variants of SARS-CoV-2 on Vero E6/TMPRSS2 cells (Table S4). Virus (∼100 PFU) was incubated with an equal volume of 2-fold-diluted serum or mAbs for 1 h. Plasma samples were diluted in calcium-free medium, while antibodies were diluted in growth medium. In addition, plasma was heat treated for 30 min at 37°C prior to use. The antibody-virus mixture was added to confluent Vero E6/TMPRSS2 cells that were plated at 30,000 cells per well the previous day in 96-well plates. The cells were incubated for 3 days at 37°C and then fixed and stained with 20% methanol and a crystal violet solution. Virus neutralization titers were determined as the reciprocal of the highest serum dilution that completely prevented cytopathic effects. The 50% inhibitory concentrations (IC_50_s) for mAbs were determined using the log(inhibitor) versus normalized response (variable slope), performed in Prism (version 9.0; GraphPad). All plasma and mAbs were tested in duplicate, and each experiment was performed twice.

### Enzyme-linked immunosorbent assay.

High-protein-binding microtiter plates (Costar) were coated with 50 μl of recombinant proteins (either full-length spike or the RBD) at 2 μg/ml in a 1× PBS solution overnight at 4°C. The plates were washed 3 times the next day with 1× PBS supplemented with 0.05% Tween 20 and blocked with 175 μl of 1× PBS containing 20% fetal bovine serum (FBS) for 1 h at 37°C. mAbs were serially diluted 1:3 starting at 10 μg/ml and incubated for 1 h at 37°C. The plates were then washed 3 times and incubated with horseradish peroxidase (HRP)-conjugated goat anti-human IgG antibody (Jackson ImmunoResearch) diluted 1:1,000 for 1 h at 37°C, and plates were subsequently developed with the Super AquaBlue enzyme-linked immunosorbent assay (ELISA) substrate (eBioscience). The absorbance was measured at 405 nm on a microplate spectrophotometer (Bio-Rad). To standardize the assays, control antibodies with known binding characteristics were included on each plate, and the plates were developed when the absorbance of the control reached 3.0 OD_405_ (optical density at 405 nm) units. All mAbs were tested in duplicate, and each experiment was performed twice.

### Competition ELISAs.

To determine the classification of certain mAbs, competition ELISAs were carried out using the mAbs with known epitope binding properties as competitor mAbs. The competitor mAbs were biotinylated overnight at 4°C with EZ-Link sulfo-NHS-biotin (Thermo Scientific). The excess free biotin of biotinylated mAbs was removed with 7,000-molecular-weight-cutoff (MWCO) Zeba spin desalted columns (Thermo Scientific). Plates were coated with 50 μl of 2 μg/ml RBD antigen overnight at 4°C. After 1 h of blocking the plates with PBS–20% FBS, a 2-fold dilution of mAbs of an undetermined class or serum was added (starting at 20 μg/ml of mAbs and a 1:50 dilution of serum) to the coated well. After incubation for 2 h at room temperature, the biotinylated competitor mAb was added at a concentration of 2× *K_d_* (dissociation constant) and incubated for another 2 h at room temperature together with mAbs or serum that was previously added. The plates were washed 3 times and incubated with 100 μl HRP-conjugated streptavidin (Southern Biotech) at a dilution of 1:1,000 for 1 h at 37°C. The plates were developed with the Super AquaBlue ELISA substrate (eBioscience). To standardize the assays, the competitor biotinylated mAb was added in a well without any competing mAbs or serum as a control well. The data were recorded when the absorbance of the control well reached 1 to 1.5 OD_405_ units. All mAbs were tested in duplicate, and each experiment was performed twice. The percent competition was then calculated by dividing a sample’s observed OD by the OD reached by the positive control, subtracting this value from 1, and multiplying by 100. For the serum data, ODs were log transformed and analyzed by nonlinear regression to determine 50% effective concentration (EC_50_) values using Prism software (version 9.0; GraphPad).

### Biolayer interferometry.

To determine the classification of certain mAbs, competition assays were performed using mAbs with known epitope binding properties as competitor mAbs with a mAb binding unknown epitopes using biolayer interferometry (BLI) with an Octet K2 instrument (Forte Bio). The RBD of SARS-CoV-2 was biotinylated, desalted, and loaded at a concentration of 10 μg/ml onto streptavidin probes for 300 s, followed by PBS for 60 s. The probe was moved to associate with mAbs of interest (10 μg/ml) for 300 s, followed by PBS for 60 s and then associations with control mAbs (10 μg/ml) for 300 s. The final volume for all the solutions was 200 ml/well. All of the assays were performed with PBS buffer at 30°C.

### SARS-CoV-2 spike and RBD protein models.

FL mutations were visualized on the WT spike protein (PDB accession number 7KJ2) using PyMOL (Schrödinger). The models of RBD mutations and RBD classes were visualized on the WT RBD protein (PDB accession number 7KDL) using PyMOL (Schrödinger). The models were further processed by Adobe Illustrator 2021 and Adobe Photoshop.

### Statistical analysis.

All statistical analyses were performed using Prism software (version 9.0; GraphPad). Sample sizes for the number of mAbs tested are indicated in the corresponding figures or in the center of pie graphs. The numbers of biological repeats for experiments and specific tests for statistical significance used are indicated in the corresponding figure legends. *P* values of ≤0.05 were considered significant (*, *P ≤ *0.05; **, *P ≤ *0.01; ***, *P ≤ *0.001; ****, *P < *0.0001).
