# Supplementary material for: Cross-Neutralization of Emerging SARS-CoV-2 Variants of Concern by Antibodies Targeting Distinct Epitopes on Spike
Source: mBio. 2021 Nov 16;12(6):e02975-21. doi: 10.1128/mBio.02975-21 (PMC8593667; doi:10.1128/mBio.02975-21)
Supplement: TABLE S1 [file mbio.02975-21-st001.docx]

**Supplementary Table 1: COVID-19 convalescent subjects.** Responder group and Severity were categorized by previous study^26^.

| **Subject ID** | **Age** | **Sex** | **SARS-CoV-2 PCR Test** | **Duration of symptoms (days)** | **Symptom start to**  **donation (days)** | **Responder Category^26^** | **Severity Category^26^** |
| --- | --- | --- | --- | --- | --- | --- | --- |
| 24 | 34 | M | 3/23/20 | 12 | 41 | High | Severe |
| 20 | 31 | M | 3/31/20 | 19 | 48 | High | Critical |
| 564 | 24 | F | 3/19/20 | 32 | 60 | Low | Severe |
| 144 | 56 | M | 3/16/20 | 23 | 54 | Low | Moderate |
| 305 | 43 | F | 4/17/20 | 4 | 47 | Low | Moderate |
| 166 | 42 | F | 3/25/20 | 17 | 55 | Low | Moderate |
| 210 | 47 | M | 4/4/20 | 7 | 41 | Low | Moderate |
| 451 | 46 | M | 4/4/20 | 11 | 49 | High | Severe (hospitalized) |
| 626 | 44 | M | 3/31/20 | 19 | 56 | High | Moderate |
| 728 | 62 | F | 3/15/20 | 53 | 130 | High | Severe |
