# Supplementary material for: Cross-Neutralization of Emerging SARS-CoV-2 Variants of Concern by Antibodies Targeting Distinct Epitopes on Spike
Source: mBio. 2021 Nov 16;12(6):e02975-21. doi: 10.1128/mBio.02975-21 (PMC8593667; doi:10.1128/mBio.02975-21)
Supplement: FIG S1 [file mbio.02975-21-sf001.docx]

**
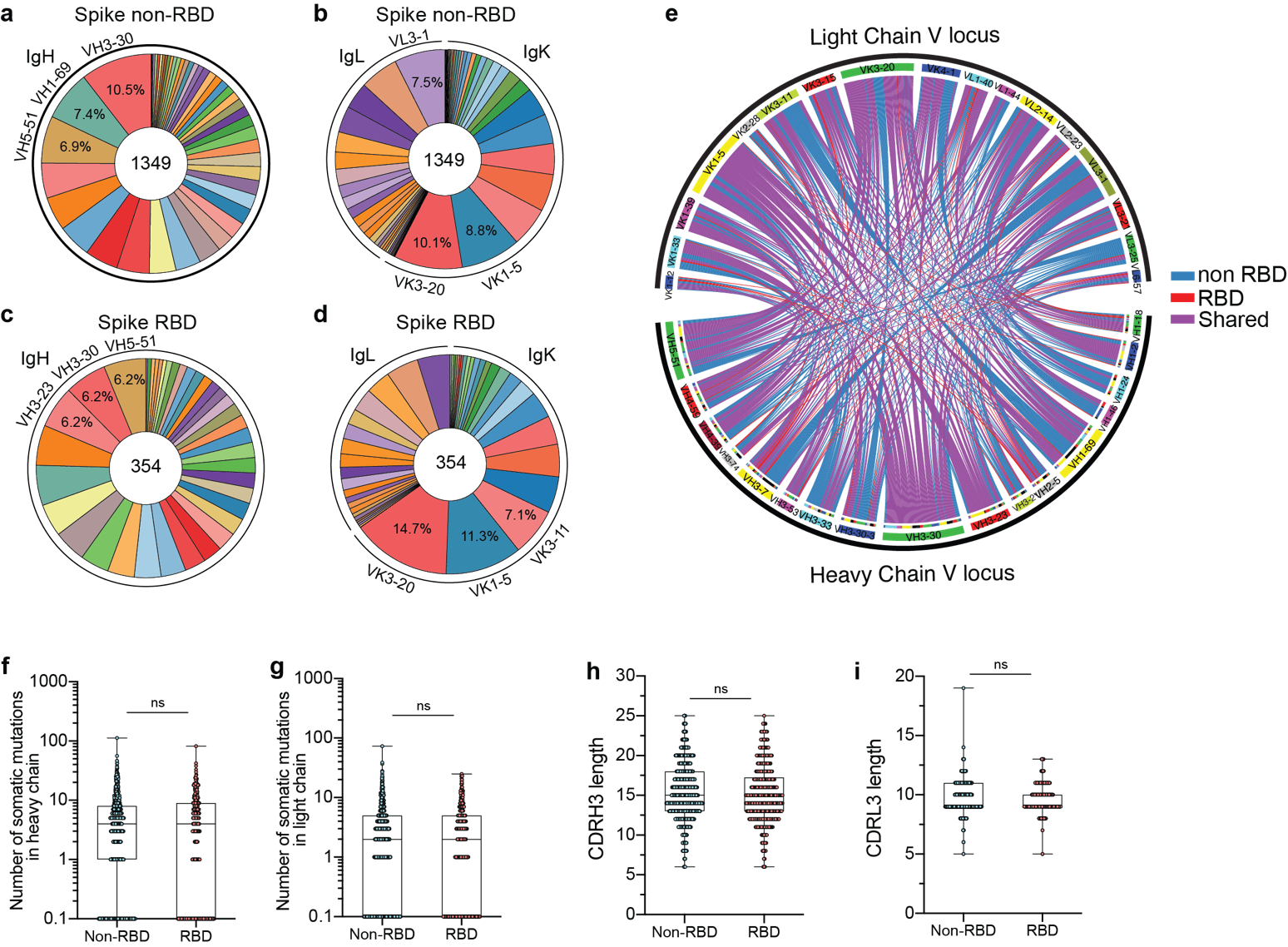
**

**Supplementary Figure 1: MAb genetic, somatic hypermutation, and CDR3 length features. a-d,** The distribution of V gene usage of spike-non RBD and spike RBD antibodies for all paired heavy **(a, c)** and light **(b, d)** chains. Percentage shown indicates proportion of the top 3 utilized genes. **e,** Clonal relationships between heavy and light chain variable gene locus of spike non-RBD and spike RBD-specific antibodies. Connecting lines represent the pairing of heavy and light chain of antibody clones specific to spike non-RBD (blue) or RBD (red) and antibody clones shared between both groups (purple). **f, g,** Comparison of number of somatic hypermutations of heavy **(f)** and light chains **(g)** of spike non-RBD and spike RBD-binding B cells. **h and i,** The complementarity determining region 3 (CDR3) amino acid length for heavy **(h)** and light chains **(i)** of spike non-RBD and spike RBD-binding B cells. Median indicated as line in the box and whisker graph. Each dot represents an individual antibody with range from minimum to maximum value. Data in **f-i** were analyzed using Mann-Whitney non-parametric test.
