# Supplementary material for: Cross-Neutralization of Emerging SARS-CoV-2 Variants of Concern by Antibodies Targeting Distinct Epitopes on Spike
Source: mBio. 2021 Nov 16;12(6):e02975-21. doi: 10.1128/mBio.02975-21 (PMC8593667; doi:10.1128/mBio.02975-21)
Supplement: TABLE S2 [file mbio.02975-21-st002.docx]

**Supplementary Table 2: Characteristics of SARS-CoV-2 spike binding mAbs.** Cross-neutralizing mAbs against WT, B.1.1.7 and P.1 or B.1.617.2 are bolded.

| **mAb ID** | **Epitope specificity** | **VH gene** | **VL gene** | **# VH SHM** | **#VL SHM** | **CDRH3 length** | **CDRL3 length** | **Database availability** |
| --- | --- | --- | --- | --- | --- | --- | --- | --- |
| S20-58 | Spike RBD  Class 2 | IGHV4-30-4*08 | IGKV2-24*01 | 5 | 2 | 15 | 9 | reference 27 |
| **S20-74** | Spike RBD  Class 3-like | IGHV4-59*11 | IGLV2-8*01 | 7 | 3 | 15 | 11 | reference 27 |
| S24-223 | Spike RBD  undetermined | IGHV2-5*02 | IGLV2-14*01 | 1 | 3 | 11 | 11 | reference 27 |
| **S24-821** | Spike RBD  Class 3 | IGHV2-70*15 | IGKV1-5*03 | 3 | 0 | 16 | 9 | reference 27 |
| S24-902 | Spike RBD  undetermined | IGHV1-69*04 | IGLV7-46*01 | 0 | 0 | 15 | 8 | reference 27 |
| S24-1002 | Spike RBD  Class 2 | IGHV3-30-3*01 | IGKV1-13*02 | 4 | 5 | 25 | 9 | reference 27 |
| **S24-1224** | Spike RBD  Class 2 | IGHV1-46*01 | IGLV1-40*01 | 8 | 7 | 20 | 11 | reference 27 |
| S24-1271 | Spike RBD  undetermined | IGHV3-66*01 | IGLV3-1*01 | 7 | 6 | 22 | 9 | reference 27 |
| S24-1301 | Spike  NTD-B | IGHV1-24*01 | IGLV10-54*01 | 6 | 4 | 18 | 11 | reference 27 |
| S24-1384 | Spike RBD  Class 4 | IGHV3-48*04 | IGLV3-21*02 | 3 | 4 | 17 | 12 | reference 27 |
| **S24-1476** | Spike RBD  Class 2 | IGHV3-49*03 | IGKV3-15*01 | 3 | 0 | 18 | 8 | reference 27 |
| S144-67 | Spike RBD  Class 3-like | IGHV5-51*01 | IGLV1-40*01 | 8 | 5 | 17 | 12 | reference 27 |
| S144-69 | Spike RBD  Class 4 | IGHV5-51*01 | IGKV1-5*01 | 3 | 3 | 11 | 8 | reference 27 |
| S144-466 | Spike RBD  Class 4 | IGHV5-51*01 | IGKV1-5*01 | 7 | 6 | 11 | 9 | reference 27 |
| S144-509 | Spike RBD  Class 4 | IGHV5-51*01 | IGKV1-5*01 | 3 | 1 | 12 | 9 | reference 27 |
| S144-1079 | Spike RBD  Class 2 | IGHV1-69*02 | IGKV3-20*01 | 9 | 3 | 19 | 9 | reference 27 |
| **S144-1339** | Spike RBD  Class 2 | IGHV1-2*06 | IGLV2-14*01 | 15 | 5 | 18 | 11 | reference 27 |
| **S144-1406** | Spike RBD  Class 2 | IGHV1-3*01 | IGKV1-5*01 | 4 | 0 | 11 | 17 | reference 27 |
| S144-1407 | Spike RBD  Class 2 | IGHV1-69*02 | IGKV1-5*01 | 9 | 2 | 12 | 10 | reference 27 |
| S144-1850 | Spike RBD  undetermined | IGHV3-23*04 | IGKV1-5*01 | 2 | 3 | 13 | 9 | reference 27 |
| **S166-32** | Spike  NTD-B | IGHV3-11*01 | IGKV1-5*01 | 10 | 2 | 19 | 8 | reference 27 |
| S166-2395 | Spike  NTD-B | IGHV4-4*07 | IGLV3-21*02 | 3 | 5 | 16 | 12 | reference 27 |
| S210-1262 | Spike  NTD-A | IGHV4-39*01 | IGLV4-69*01 | 11 | 4 | 10 | 9 | reference 27 |
| S305-223 | Spike RBD  Class 2 | IGHV3-33*06 | IGKV3-11*01 | 18 | 8 | 6 | 9 | reference 27 |
| S305-399 | Spike RBD  undetermined | IGHV1-24*01 | IGKV3-15*01 | 4 | 4 | 18 | 9 | reference 27 |
| **S305-1456** | Spike  NTD-B | IGHV1-24*01 | IGKV3-15*01 | 3 | 3 | 20 | 9 | reference 27 |
| S451-11 | Spike  NTD-A | IGHV3-23*01 | IGKV3D-20*01 | 8 | 3 | 15 | 8 | internal data |
| S451-337 | Spike  NTD-B | IGHV4-59*01 | IGKV3-20*01 | 10 | 1 | 15 | 10 | internal data |
| S451-650 | Spike S2 | IGHV3-30*01 | IGKV3-20*01 | 6 | 4 | 14 | 8 | internal data |
| S451-1451 | Spike  NTD-A | IGHV4-31*01 | IGLV2-11*01 | 9 | 5 | 14 | 10 | internal data |
| S451-1522 | Spike  NTD-B | IGHV2-26*01 | IGLV2-14*01 | 8 | 5 | 20 | 11 | internal data |
| S564-14 | Spike RBD  Class 3-like | IGHV3-7*01 | IGLV3-21*04 | 6 | 3 | 18 | 12 | reference 27 |
| **S564-68** | Spike RBD  Class 2 | IGHV1-2*02 | IGLV2-8*01 | 6 | 2 | 15 | 10 | reference 27 |
| **S564-134** | Spike RBD  Class 2 | IGHV1-2*02 | IGLV2-8*01 | 2 | 6 | 15 | 10 | reference 27 |
| S564-138 | Spike RBD  Class 2 | IGHV1-2*02 | IGLV2-14*01 | 10 | 1 | 18 | 10 | reference 27 |
| S564-152 | Spike RBD  Class 4 | IGHV3-33*06 | IGKV1-33*01 | 4 | 4 | 20 | 10 | reference 27 |
| **S564-265** | Spike RBD  Class 2 | IGHV1-2*02 | IGLV2-8*01 | 4 | 3 | 15 | 10 | reference 27 |
| S626-8 | Spike S2 | IGHV1-8*01 | IGLV3-19*01 | 7 | 5 | 24 | 12 | this study |
| S626-362 | Spike RBD  undetermined | IGHV3-48*01 | IGLV1-40*01 | 18 | 3 | 16 | 11 | this study |
| S626-651 | Spike RBD  Class 3-like | IGHV1-69*04 | IGLV1-40*01 | 6 | 4 | 17 | 11 | this study |
| **S626-747** | Spike RBD  Class 3-like | IGHV3-9*01 | IGKV1-33*01 | 6 | 6 | 22 | 10 | this study |
| S728-1981 | Spike  NTD-A | IGHV1-46*01 | IGKV3-11*01 | 17 | 3 | 16 | 11 | this study |
| S728-2036 | Spike  NTD-A | IGHV1-2*02 | IGLV2-23*02 | 14 | 12 | 17 | 10 | this study |
