# Supplementary material for: Cross-Neutralization of Emerging SARS-CoV-2 Variants of Concern by Antibodies Targeting Distinct Epitopes on Spike
Source: mBio. 2021 Nov 16;12(6):e02975-21. doi: 10.1128/mBio.02975-21 (PMC8593667; doi:10.1128/mBio.02975-21)
Supplement: FIG S2 [file mbio.02975-21-sf002.docx]

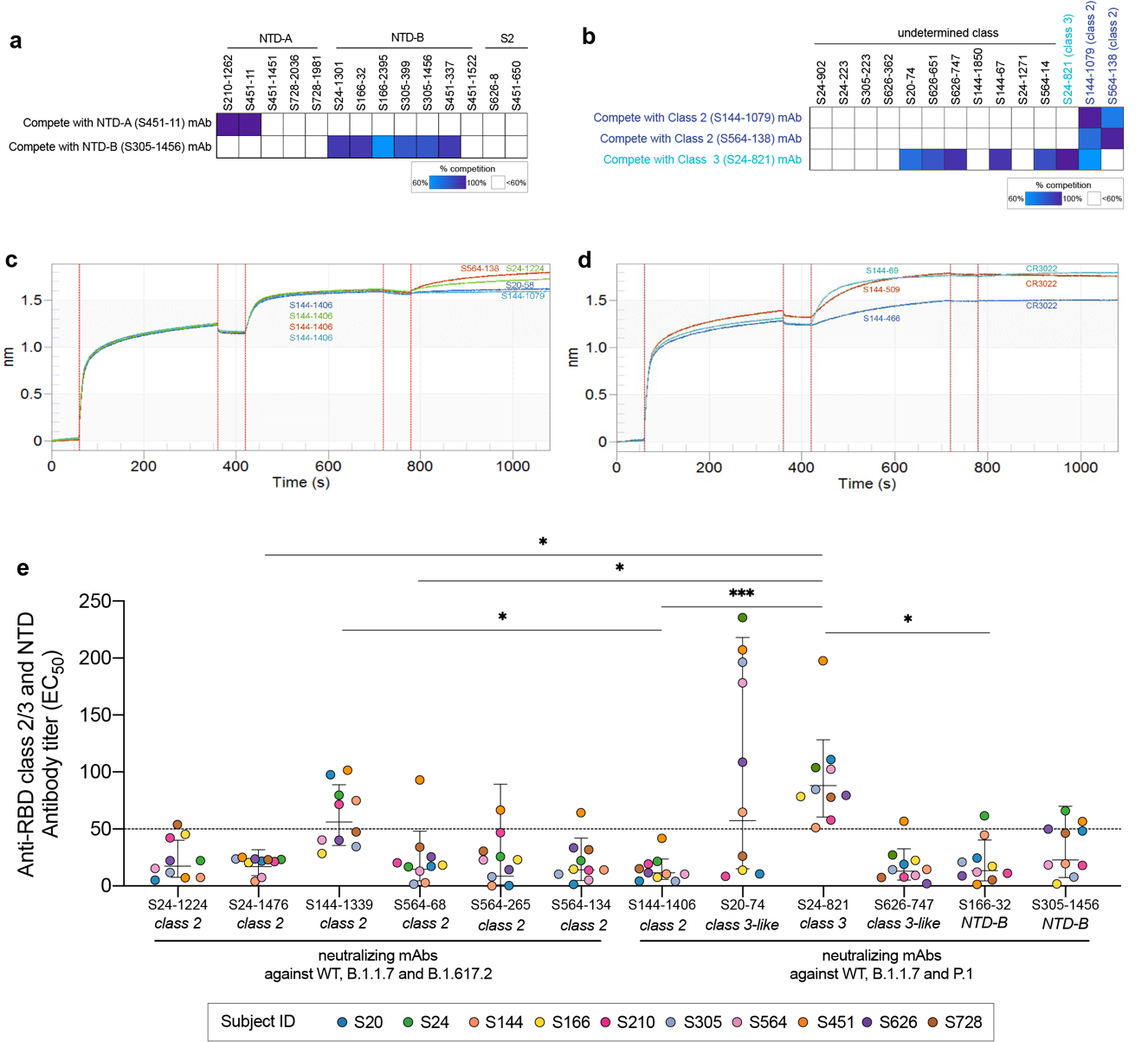


**Supplementary Figure 2: MAb binding competition by ELISA and BLI and serum competition by ELISA. a,** Competition ELISA of RBD mAbs of spike non-RBD mAbs with NTD-A (S451-11) and NTD-B (S305-1456). **b,** Competition ELISA of RBD mAbs of undetermined class with class 2 mAbs (S144-1079 and S564-138) and class 3 mAb (S24-821). **c,** MAb binding competition by BLI of class 2 mAb, S144-1406, with the other class 2 mAbs (n=4) that did not neutralize P.1. **d,** MAb binding competition by BLI between class 4 mAbs that utilized VH5-51 (S144-466, S144-509, S144 and S144-69) with CR3022. **e**, EC_50_ of serum antibodies of 10 convalescent subjects competing with RBD-reactive mAbs for binding to RBD class 2, class 3 and class 3-like epitopes, and NTD-reactive mAbs for binding to NTD-B epitopes. Dashed line represents the limit of detection**.** Data in **a**-**b** and **e** are representative of two independent experiments performed in duplicate. Data in **e** were analyzed using nonparametric Friedman's test with Dunn’s multiple comparison test.
