# Supplementary material for: Cross-Neutralization of Emerging SARS-CoV-2 Variants of Concern by Antibodies Targeting Distinct Epitopes on Spike
Source: mBio. 2021 Nov 16;12(6):e02975-21. doi: 10.1128/mBio.02975-21 (PMC8593667; doi:10.1128/mBio.02975-21)
Supplement: TABLE S4 [file mbio.02975-21-st004.docx]

**Supplementary Table 4: SARS-CoV-2 virus information and source.**

| **Antigen** | **S1 NTD** | **RBD** | **S1 CTD** | **S2** | **Source** |
| --- | --- | --- | --- | --- | --- |
| WT | - | - | - | - | SARS-CoV-2/UT-NCGM02/Human/2020/Tokyo from BEI |
| B.1.1.7 | L5F, H69del, V70del, Y144del | N501Y | A570D, D614G, P681H | T716I, S982A, D1118H | hCoV-19/Japan/QHN001/2020 from BEI |
| P.1 | L18F, T20N, P26S, D138Y, G181V, R190S | K417T, E484K, N501Y | D614G, H655Y | T1027I, V1176F | hCoV-19/Japan/TY7-501/2021 from BEI |
| B.1.617.1 | G142D, E154K | L452R, E484Q | D614G, P681R | Q1071H, H1101D | hCoV-19/USA/CA-Stanford-15_S02/2021 from BEI |
| B.1.617.2 | T19R, T95I, G142D, E156G, F157del, R158del | L452R, T478K | D614G, P681R | D950N | hCoV-19/USA/WI-UW-5250/2021 |
