# Supplementary material for: Cross-Neutralization of Emerging SARS-CoV-2 Variants of Concern by Antibodies Targeting Distinct Epitopes on Spike
Source: mBio. 2021 Nov 16;12(6):e02975-21. doi: 10.1128/mBio.02975-21 (PMC8593667; doi:10.1128/mBio.02975-21)
Supplement: TABLE S3 [file mbio.02975-21-st003.docx]

**Supplementary Table 3: Antigen information and source.** VOC refers to variant of concern and VUM refers to variant under monitoring.

| **Antigen** | **S1 NTD** | **RBD** | **S1 CTD** | **S2** | **Mutation detected in** | **Source** |
| --- | --- | --- | --- | --- | --- | --- |
| Spike FL, 2-P, trimer | | | | | |  |
| WT | - | - | - | - | - | In-house |
| D614G | - | - | D614G | - | VOC | In-house |
| B.1.1.7 (alpha) | H69del, V70del, Y144del | N501Y | A570D, D614G, P681H | T716I, S982A, D1118H | VOC | Sather lab |
| B.1.351 (beta) | L18F, D80A, D215G, del241-243, R246I | K417N, E484K, N501Y | D614G | A701V | VOC | Sather lab |
| P.1 (gamma) | L18F, T20N, P26S, D138Y, R190S | K417T, E484K, N501Y | D614G, H655Y | T1027I, V1176F | VOC | Sather lab |
| B.1.617.2 (delta) | T19R, G142D, del156-157, R158G | L452R, T478K, | D614G, P681R | D950N | VOC | Sather lab |
| B.1.526 (iota) | L5F, T95I, D253G | E484K | D614G | A701V | VUM | Sather lab |
| B.1.617.1 (kappa) | T95I, G142D, E154K | L452R, E484Q | D614G, P681R | Q1071H | VUM | Sather lab |
| S1 monomeric | | | | | |  |
| WT | - | - | - | - | - | SinoBiological |
| S2 monomeric | | | | | |  |
| WT | - | - | - | - | - | SinoBiological |
| RBD | | | | | |  |
| WT | - | - | - | - | - | In-house |
| E406Q | - | E406Q | - | - | Circulating variant*, In vitro* escape | Krammer lab |
| K417N (B.1.351) | - | K417N | - | - | VOC, *In vitro* escape | SinoBiological |
| K417T (P.1) | - | K417T | - | - | VOC, *In vitro* escape | In-house |
| K417E | - | K417E | - | - | *In vitro* escape | Krammer lab |
| K417V | - | K417V | - | - | *In vitro* escape | Krammer lab |
| K417A | - | K417A | - | - | RBD-ACE2 contacting | In-house |
| Y453F (B.1.427, B.1.429) | - | Y453F | - | - | VOC, *In vitro* escape | Krammer lab |
| F486A | - | F486A | - | - | *In vitro* escape | Krammer lab |
| N487R | - | N487R | - | - | *In vitro* escape | Krammer lab |
| E484K (P.1, B.1.526, B.1.351, B.1.1.318, B.1.525, R.1, B.1.526.2, B.1.1, B.1.621, B.1, B.1.1.7) | - | E484K | - | - | VOC, *In vitro* escape | Krammer lab |
| F490K | - | F490K | - | - | *In vitro* escape | Krammer lab |
| Q493R | - | Q493R | - | - | *In vitro* escape | Krammer lab |
| N439K | - | N439K | - | - | VOC, *In vitro* escape | Krammer lab |
| N440K (B.1.36) | - | N440K | - | - | Circulating variant, *In vitro* escape | Krammer lab |
| L452R (B.1.526.1, B.1.429, B.1.427, B.1.617.2, B.1, B.1.617.1, C.36, A.2.5) | - | L452R | - | - | VOC, *In vitro* escape | SinoBiological |
| N501Y (B.1.1.7) | - | N501Y | - | - | VOC | Krammer lab |
| N501Q | - | N501Q | - | - | RBD-ACE2 contacting | Krammer lab |
| N501A | - | N501A | - | - | RBD-ACE2 contacting | Krammer lab |
| B.1.351 |  | K417N, E484K, N501Y |  |  | VOC | Krammer lab |
| P.1 |  | K417T, E484K, N501Y |  |  | VOC | Krammer lab |
| Other coronaviruses | | | | | |  |
| SARS-CoV-1 RBD WT | | | | | - | In-house |
| MERS-CoV RBD WT | | | | | - | In-house |
