# Supplementary material for: Cross-Neutralization of Emerging SARS-CoV-2 Variants of Concern by Antibodies Targeting Distinct Epitopes on Spike
Source: mBio. 2021 Nov 16;12(6):e02975-21. doi: 10.1128/mBio.02975-21 (PMC8593667; doi:10.1128/mBio.02975-21)
Supplement: FIG S3 [file mbio.02975-21-sf003.docx]

**
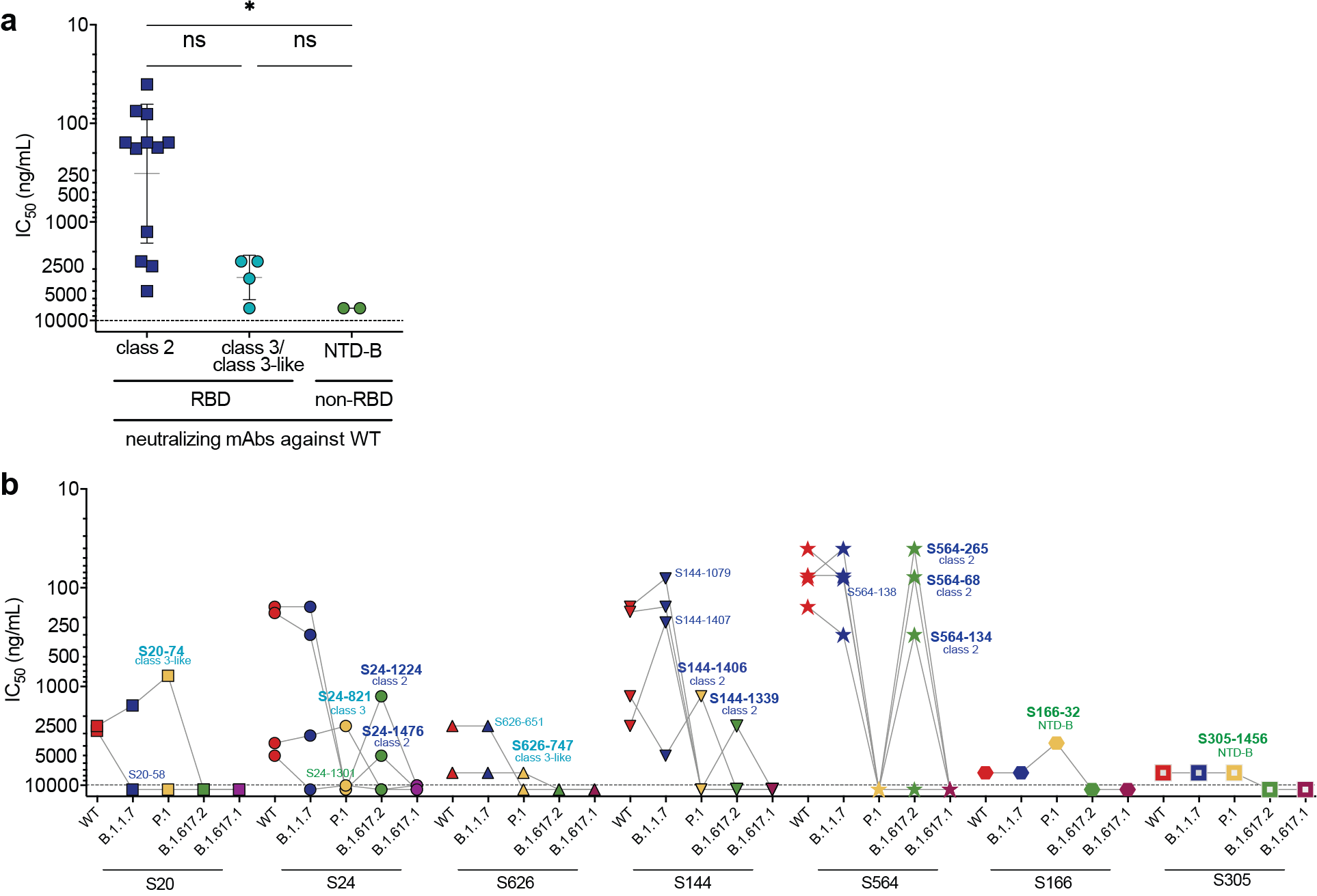
**

**Supplementary Figure 3: Comparison of neutralization potency of SARS-CoV-2 neutralizing mAbs. a,** Neutralization potency (IC_50_) of RBD-binding mAbs, class 2 and class 3, and NTD-B binding mAbs against WT SARS-CoV-2. **b,** Neutralization potency of each mAb from each subject against WT SARS-CoV-2 (red), B.1.1.7 (blue), P.1 (yellow), B.1.617.1 (green) and B.1.617.2 (plum). Each dot indicates one mAb. MAbs that neutralize VOCs are bolded. Data in **a**-**c** are representative of two independent experiments performed in duplicate. Data in **a** were analyzed using Mann-Whitney non-parametric test.
